# Microwave-Hydrothermal Treated Grape Peel as an Efficient Biosorbent for Methylene Blue Removal

**DOI:** 10.3390/ijerph15020239

**Published:** 2018-01-31

**Authors:** Lin Ma, Chunhai Jiang, Zhenyu Lin, Zhimin Zou

**Affiliations:** 1 Department of Industry and Academy Collaborative Development, Xiamen University of Technology, 600 Ligong Road, Jimei District, Xiamen 361024, China; lma@xmut.edu.cn; 2 Fujian Provincial Key Laboratory of Functional Materials and Applications, Institute of Advanced Energy Materials, School of Materials Science and Engineering, Xiamen University of Technology, 600 Ligong Road, Jimei, Xiamen 361024, China; 3 MOE Key Laboratory of Analysis and Detection for Food Safety, Fujian Provincial Key Laboratory of Analysis and Detection Technology for Food Safety, Institute of Nanomedicine and Nanobiosensing, College of Chemistry, Fuzhou University, Fuzhou 350116, China; zylin@fzu.edu.cn

**Keywords:** grape peel, biosorbent, biosorption, methylene blue, wastewater treatment

## Abstract

Biosorption using agricultural wastes has been proven as a low cost and efficient way for wastewater treatment. Herein, grape peel treated by microwave- and conventional-hydrothermal processes was used as low cost biosorbent to remove methylene blue (MB) from aqueous solutions. The adsorption parameters including the initial pH value, dosage of biosorbents, contact time, and initial MB concentration were investigated to find the optimum adsorption conditions. The biosorbent obtained by microwave-hydrothermal treatment only for 3 min at 180 °C (microwave-hydrothermal treated grape peel, MGP) showed faster kinetics and higher adsorption capability than that produced by a conventional-hydrothermal process (hydrothermal treated grape peel, HGP) with a duration time of 16 h. The maximum adsorption capability of MGP under the optimum conditions (pH = 11, a dosage of 2.50 g/L) as determined with the Langmuir model reached 215.7 mg/g, which was among the best values achieved so far on biosorbents. These results demonstrated that the grape peel treated by a quick microwave-hydrothermal process can be a very promising low cost and efficient biosorbent for organic dye removal from aqueous solutions.

## 1. Introduction

Dyes are widely used in textile, paper, rubber, plastics, cosmetics, printing, and dyeing processes, which produce a huge amount of dye-containing wastewater every year, and may thus bring much harm to human beings. Taking methylene blue (MB) as an instance, it can cause many symptoms to people who inhale or ingest MB orally, such as short periods of rapid or difficult breathing, burning sensation, nausea, vomiting, diarrhea, and gastritis [1]. Because of their synthetic origin and complex aromatic structures, dyes are not easily biodegraded [2]. Therefore, the effluents of dye-processing industries must be well treated before they are released to alleviate their contamination to the natural water system. 

To date, the methods used for organic dye removal include coagulation and flocculation [2], biological oxidation, chemical precipitation [3], photocatalytic degradation [4,5], and adsorption [6,7,8,9,10]. Among them, adsorption is one of the most effective and convenient ways. Unfortunately, the most commonly used industrial adsorbent, activated carbon, is too expensive for industrial-scale applications even though it shows high adsorption efficiency and is wide available. Therefore, seeking alternative low-cost adsorbents other than activated carbons in combination with tuning the chemical properties of organic dyes towards more economic and efficient dye removal has seen increasing interest in the water cleaning processes [11,12]. In fact, both strategies fulfill the basic innovation principles of the theory of inventive problem solving (TRIZ) [13].

Recently, agricultural wastes or byproducts—such as hazelnut shells [14], sawdust [15], wheat shells [1], garlic peel [16], and rice husbk [3], and raw pomegranate peel [17], etc.—have been investigated as low-cost biosorbents for dye removal. The biosorbents contain proteins, polysaccharides, and lignin, which are associated with functional groups responsible for pollution adsorption [18]. The abundant natural occurrence and presence of a large amount of surface functional groups make the agricultural wastes good adsorbents for organic dye molecules [12]. As a major fruit crop worldwide, grapes have a harvest amount of about 60 million tons per year, and 20 wt % of that becomes biomass waste. As an instance, wine industry generates about 5–9 million tonnes of grape waste per year worldwide [19]. The grape waste is rich in polyphenol compounds, which can cause many detrimental effects on the flora and fauna of discharged zones [20]. Therefore, using grape waste as a biosorbent can solve not only the problem of toxic effluents but also the disposal problem of this biomass waste. R. Chand and coauthors prepared an adsorbent gel from grape waste by crosslinking with concentrated sulfuric acid, which showed the maximum adsorption capacity of 1.91 moL/kg for chromium ions (VI) [21]. Giuseppina Avantaggiato et al. [22] reported that grape pomace (pulp and peels) was able to rapidly sequester and different mycotoxins simultaneously, and thus could be used as an efficient biosorbent for removing mycotoxins from liquid media. Dried grape pulp was also able to uptake MB of 78.74, 116.28, and 153.85 mg/g at temperatures of 20 °C, 40 °C, and 60 °C, respectively [23].

The preparation of grape waste biosorbent usually requires dehydration using concentrated sulfuric acid or direct drying [21,23]. The former will result in additional waste water that is difficult to dispose. The latter requires a long time as the grape pulp is difficult to be completely dried and grounded. In this study, microwave-hydrothermal treatment was performed rapidly dehydrate grape peels as compared to the time-consuming conventional hydrothermal process. After drying, the adsorption property of the thus-prepared biosorbents was tested by removing methylene blue (MB) from aqueous solution both isothermally and kinetically. The adsorption parameters—including the initial pH value, dosage of biosorbents, contact time and initial MB concentration—were investigated to find the optimum adsorption conditions. The biosorbents, especially the one prepared by microwave-hydrothermal process, exhibited outstanding removal ability of MB as compared to many other biosorbents reported recently.

## 2. Materials and Methods

### 2.1. Preparation and Characterizations of Grape Peel Biosorbents

The grape peel was obtained by manually peeling Kyoho grapes purchased from a local market. After cleaning with deionized water, the grape peel was squeezed to get rid of the water. A certain amount of the wet grape peel was loaded into a Teflon-lined nylon autoclave, which was installed in microwave-processing equipment (Michem MD6C-6H, Beijing, China) and heated at 180 °C for 3 min. After cooling, the treated grape peel was filtered out, washed by ultrapure water repeatedly and dried at 80 °C. For comparison, grape peel biosorbent was also prepared by conventional hydrothermal treatment at 180 °C for 16 h with the other conditions unchanged. The dried grape peel was manually grinded into powders and then sieved to 60 mesh particles to obtain the biosorbents. To simplify the description, the grape peel biosorbents prepared by microwave-hydrothermal treatment and conventional-hydrothermal treatment were denoted as MGP and HGP, respectively. 

The surface functional groups of the biosorbents were analyzed using a Fourier-transform infrared spectrometer (FTIR, Alpha, Bruker, Germany). The Brunauer-Emmett-Teller (BET) surface areas were measured using a pore size and surface area equipment (Nova 2200e, Quantachrome Instruments, Boynton Beach, FL, USA). The microstructural morphologies were observed by a scanning electronic microscope (SEM, Zeiss EVO 18, Carl Zeiss, Gemerny).

### 2.2. Adsorption Experiments

Cationic methylene blue was chosen as a model dye adsorbate in this study. The MB with a chemical formula of C_16_H_18_CN_3_S and molecular mass of 319.86 g/moL was purchased from Aladdin Company and used as received. A MB stock solution of 1 g/L was prepared by dissolving MB in ultrapure water (18.2 MΩ). The adsorption experiments were carried out using a batch test model. The influence of initial solution pH on MB removal was studied over a pH range of 3.5–11.52. The pH values of the MB solution (20 mL, 150 mg/L, and 500 mg/L) were adjusted by adding negligible volume of 0.1 M NaOH or 0.1 M HCl solutions. 50 mg biosorbents were agitated in the MB solutions for 2 h. The suspension was filtered and the residual concentrations of MB in the filtrates were measured using an UV–visible spectrometer (UV-1200, Mapada, Shanghai, China) taking 665 nm as the collaboration wavelength. The adsorbed amounts of MB were calculated according to Equation (1)

*q*_*e*_ = (*C*_0_ − *C*_*e*_)*V*/*m* (1)

where *C*_0_ is the initial concentration, *C_e_* is the equilibrium concentration, *V* is the volume of the liquid phase, and *m* is the mass of the adsorbent.

The effect of biosorbent dosage on MB removal was studied by agitating different mass of biosorbent in 20 mL solution with an initial MB concentration of 500 mg/L. The adsorption isotherms were obtained by agitating 50 mg of each kind of biosorbent in 20 mL MB solutions with varied initial concentrations (100, 200, 300, 400, 500, and 600 mg/L) at 240 rpm for 3 h at room temperature (25 °C). The pH value was set at about 11.2. To evaluate the adsorption kinetics, 150 mg of the biosorbents were dispersed in 60 mL of MB solution (600 mg/L) and agitated at 240 rpm. At certain time intervals, 5 mL of the solution mixture was extracted out and subjected to filtration and MB concentration analysis. The adsorption amounts were calculated following Equation (2)

*q*_*t*_ = (*C*_0_ − *C*_*t*_)*V*/*m* (2)

where *C*_0_ is the initial concentration, *Ct* is the remaining concentration at time *t*, *V* is the volume of the liquid phase, and m is the mass of the biosorbents. All adsorption experiments were carried out at least three times and the averaged data was presented in this manuscript.

## 3. Results and Discussion

### 3.1. Characterizations of the Biosorbents

**Figure 1:**
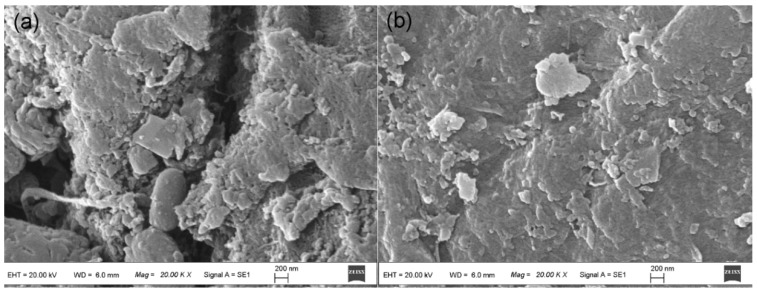
SEM images of the biosorbents, (**a**) HGP and (**b**) MGP.

The microstructures of the as-prepared biosorbents were examined by SEM. Figure 1 shows the SEM images of HGP (a) and MGP (b) biosorbents. As can be seen, both samples show sheet-like microstructure assembled by nanoscale subunits, which might originate from the natural structure of the grape peel. The HGP sample exhibits a little bit more porous structure than MGP, possibly due to the longer hydrothermal treatment period.

**Figure 2:**
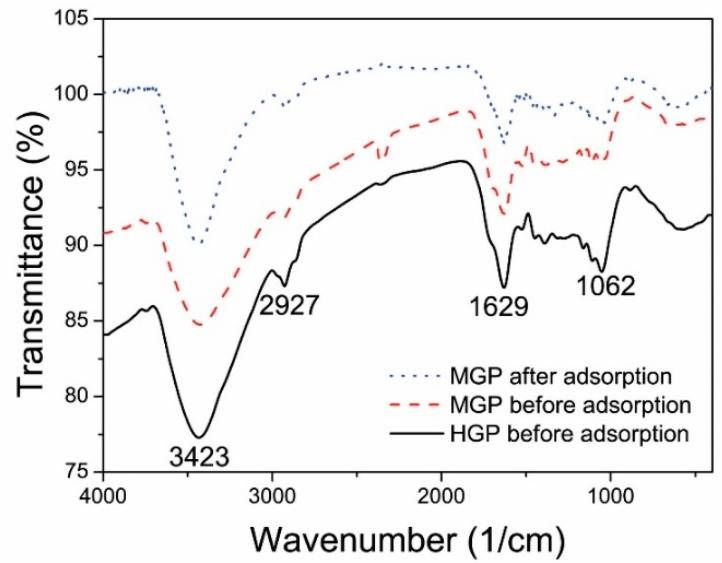
FTIR spectra of HGP before MB adsorption and MGP before and after MB adsorption.

The surface functional groups of the biosorbents were characterized by FTIR spectra. Figure 2 shows the FTIR spectrum of HGP before MB sorption and those of MGP before and after MB adsorption, respectively. The peak located at about 3423 cm^−1^ is due to the stretch vibrating of –OH groups [3]. The peak at 2927 cm^−1^ can be ascribed to C–H stretching of the methylene groups [24]. The bands corresponding to C=O stretching of carboxyl group (1637 cm^−1^) and C–O stretching of alcohols (1062 cm^−1^) are also clearly visible. However, as compared to other biomasses [24], the bands at 2850 cm^−1^ and 1413 cm^−1^ that correspond to the C–H stretching of aromatic groups and –OH stretching of phenols disappear, indicating that the polyphenols in the grape peel have been decomposed during the hydrothermal treatments. The results show that the hydrothermally treated grape peel biosorbents contain several kinds of functional groups such as hydroxyl, alcohol, and carboxyl, which may be adsorption sites for MB. However, as clearly shown in Figure 2, the FTIR spectrum of MGP does not change much after MB adsorption, indicating that the adsorption of MB does not change the surface chemical properties of the biosorbent.

The BET surface areas and total pore volumes of both biosorbents as determined by N_2_ adsorption/desorption measurements at 77 K are listed in Table 1. As can be seen, both biosorbents show low BET surface areas and small pore volumes. As will be discussed below, this low surface area cannot account for the large adsorption amount of MB. It is believed that the adsorption of MB should be mainly processed by its interactions with the surface functional groups of the biosorbents.

### 3.2. Adsorption Properties

#### 3.2.1. Effect of Solution pH

**Figure 3:**
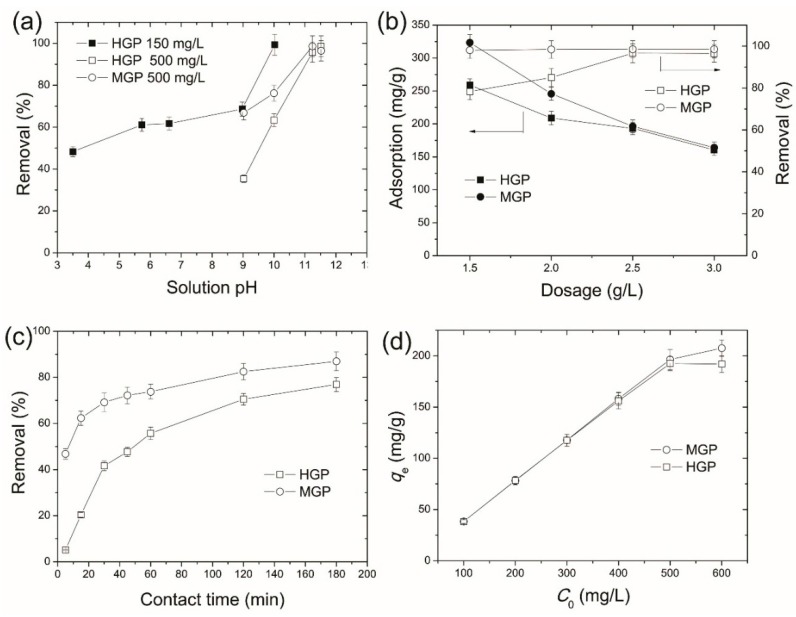
Effect of (**a**) initial solution pH, (**b**) dosage, (**c**) contact time (feed pH of 11, initial MB concentration of 600 mg/L, room temperature), and (**d**) initial solution concentrations on the removal of MB (feed pH of 11, dosage of 2.5 g/L, room temperature) on grape-peel based biosorbents.

The pH of aqueous solution is an important parameter affecting the interaction between the dye molecular and biosorbents. Therefore, the initial pH of MB solution was mediated first to examine the adsorption abilities of the grape peel biosorbents. Figure 3a shows the variation of removal efficiencies of HGP and MGP towards MB as a function of solution pH. At a MB concentration of 150 mg/L, the percentage removal of MB on HGP increases from 48.3% to 99.3% when the pH is increased from 3.5 to 10.0. In addition, at a MB concentration of 500 mg/L, the removal rates of both the MGP and HGP biosorbents reach about 98.6% at pH value of 11.2 or 11.5. This clearly indicates that a basic condition is more favorable for MB adsorption on the grape peel biosorbents. This can be explained by the cationic nature of MB molecules, which create positively charged ions when dissolved in water, and the positively charged surface of biosorbent in acidic medium (lower pH) will therefore tend to oppose the adsorption of the cationic dye. As the pH of dye solution is increased, the surface of the biosorbent acquires a negative charge, which enhances the electrostatic attraction between the negatively charged biosorbents and the positively charged dye, resulting in increased MB adsorption [25]. A similar trend was observed for adsorption of methylene blue onto *Platanus orientalis* leaf powder [25], yellow passion fruit waste [26], and Brazil nut shells [27]. According to the results shown in Figure 3a, an initial pH value of about 11 ± 0.2 was set for the following adsorption experiments.

#### 3.2.2. Effect of Dosage

The effect of biosorbent dosage on the equilibrium adsorption capabilities and removal efficiencies of MB is depicted in Figure 3b. For MGP, the removal of MB is higher than 98% when the dosage is larger than 1.5 g/L even when the starting MB concentration is as high as 500 mg/L. Further increasing the dosage to 2.5 g/L, the removal only slightly increases to 98.5%. By contrast, the removal of MB on HGP is only 78.3% at a dosage of 1.5 g/L. Further increasing the dosage to 2.5 g/L the removal efficiency reaches 96.6%. This indicates that the MGP owns obviously higher adsorption capability than HGP towards MB uptake. From Figure 3b we can also see that increasing the dosage almost linearly decreases the adsorption capabilities per unit mass of the biosorbents. Therefore, generally considering the removal efficiency and usage of the biosorbents, a dosage of 2.5 g/L, i.e., 50 mg biosorbent in 20 mL MB solution, was applied in the following adsorption experiments.

#### 3.2.3. Effect of Contact Time

The effect of contact time on the adsorption process is shown in Figure 3c. The adsorption process can be basically divided into two stages, i.e., a rapid initial adsorption stage and a second slow adsorption stage. For an initial concentration of 600 mg/L, MGP can remove 46.8% of the total MB after only 5 min, whereas HGP only uptakes 5.1% of the total MB within the same time. The adsorption of MB on MGP reaches the second slow stage after 30 min, followed by adsorption equilibrium at 180 min, with a total removal of 87%. However, although the adsorption of MB on HGP also reaches the second stage after 30 min, the adsorption keeps increasing thereafter and an equilibrium has not yet been obtained after 180 min (with a removal of 76.9%). This clearly demonstrates that the MGP not only possesses faster adsorption kinetics, but also higher adsorption capability than HGP towards MB removal. The rapid initial adsorption is due to the fact that all adsorbent sites are vacant and the solute concentration gradient is very high. The decrease in the adsorption rate toward the end of experiment is due to the decreased number of vacant sites of biosorbents and dye concentrations [25]. This also hints that the adsorption may be processed through monolayer formation of MB molecules on the adsorbent surface.

#### 3.2.4. Effect of Initial MB Concentration

The effect of initial MB concentration on adsorption is shown in Figure 3d. It is evident that *q_e_* increases with the increase of initial MB for both biosorbents. The amounts of MB adsorbed at equilibrium increase from 38.4 to 207.6 mg/g on MGP and from 38.3% to 192.5 mg/g on HGP, respectively, when the initial dye concentration increases from 100 to 600 mg/L. It is supposed that the mass transfer driving force enhances when the initial dye concentration increases, resulting in a high adsorption of MB [25]. From Figure 3d, one can also extrapolate that, for an initial MB concentration of 600 mg/L, the adsorption by MGP leaves lower equilibrium concentration of MB in the residue solution. In other words, MGP has higher removal ability towards MB than HGP.

#### 3.2.5. Adsorption Isotherms

The adsorption isotherms are investigated by fitting the experimental data with Langmuir isotherm model by linearly plotting *C_e_/q_e_* against *C_e_* (Equation (3)) or Freundlich isothermal model by linearly plotting ln *q_e_* against ln *C_e_* (Equation (4)), respectively, as shown in Table 2.

*C*_*e*_/*q*_*e*_ = *k*_3_/*q*_*m*_ + *C*_*e*_/*q*_*m*_ (3)

ln *q*_*e*_ = ln *k*_4_ + *n*^−1^ ln *C*_*e*_ (4)

where *C_e_* is the equilibrium concentration of MB (mg/L), *q_e_* is the equilibrium adsorption capacity (mg/g), *q_m_* (mg/g) and *k*_3_ (L/mg) are the maximum adsorption capacity and the equilibrium constant, respectively, and *k*_4_ and *n* are the constants of Freundlich adsorption.

The treatment by Freundlich isotherm model yields a poor correlation coefficient of *R*^2^ = 0.189 and 0.454 for MGP and HGP, respectively. This indicates that the adsorption does not follow a multilayer formation mechanism. By contrast, a correlation coefficient (*R*^2^) of higher than 0.99 is obtained for the fitting to Langmuir isotherm model, suggestive of monolayer formation of the adsorbate on the surface of biosorbents. The maximum adsorption amounts of MB on MGP and HGP, as estimated by Langmuir simulation, are 215.7 and 192.7 mg/g, respectively, which are among the best adsorption properties reported so far for biosorbents [25].

#### 3.2.6. Adsorption Kinetics

The pseudo-first order (Equation (5)) and pseudo-second order (Equation (6)) kinetic models were used to fit the experimental data.

ln (*q_e_* − *q_t_*_)_ = ln *q_e_* − *k*_5_*t* (5)

*t*/*q_t_* = 1/(*k*_6_*q_e_*^2^) + *t*/*q_e_* (6)

where *q_e_* is the adsorption capacity (mg/g) at equilibrium, *q_t_* is the adsorption capacity at time *t*, *k*_5_ (1/min) is the pseudo-first-order adsorption rate constant, *k*_6_ is the initial adsorption rate (mg/g/min). The fitted kinetics data based on the pseudo-first and pseudo-second order models are listed in Table 3. Based on the high correlation coefficients, both the pseudo-first order and pseudo-second order kinetic models can effectively describe the MB adsorption behavior on both MGP and HGP biosorbents. This illustrates that the adsorption of MB on the prepared biosorbents is controlled by both physisorption and chemisorption processes. The *k*_6_ value of HGP is smaller than that of MGP, indicating that the initial adsorption rate of MB on the former is slower, consistent with the results shown in Figure 3c.

Based on the above results, we can postulate that the adsorption mechanism of MB on the grape peel biosorbents may proceed by both physisorption and chemisorption processes, which may go through formation of monolayer MB molecules on the surface of biosorbents.

#### 3.2.7. Comparison of Various Adsorbents

To illustrate the superior adsorption ability of the grape peel biosorbents for MB, the maximum monolayer adsorption capacity of the MGP in this work is compared to some other biosorbents, as shown in Table 4. As observed, the maximum monolayer adsorption capacity of MB on MGP (*q_m_* = 215.7 mg/g) is much higher than those of most recently reported biosorbents, indicating the promising application of MGP for organic dye removal. For example, the dried grape pulp can uptake 118.3 mg/g of MB, but this value is still much lower than the maximum adsorption capacity of 215.7 mg/g achieved on the MGP biosorbent prepared in this work. Further considering the quick microwave-hydrothermal process for the biosorbent preparation, our work presented a potential grape-peel based low-cost biosorbent as well as its fast and simple fabrication method for efficient organic dye removal.

## 4. Conclusions

Biosorbents prepared from grape peel by microwave-assisted and conventional hydrothermal treatments were used to remove methylene blue from aqueous solutions. The adsorption showed higher capability at higher pH value because of the cationic nature of MB. Although the biosorbents obtained by microwave-assisted and conventional hydrothermal treatments possessed similar morphology and surface functional groups, the former showed obviously faster kinetics and higher adsorption capability than the latter. A high adsorption capability (*q_m_*) of 215.7 mg/g was determined with the Langmuir model on MGP under the optimum conditions (pH = 11, a dosage of 2.50 g/L). This high adsorption capacity and the quick process for biosorbent preparation demonstrated that the grape peel prepared by microwave-hydrothermal treatment can be a very promising low cost and efficient biosorbent for methylene blue removal. The prepared biosorbent may be used to remove other organic dyes or heavy metal ions from wastewater. The microwave-hydrothermal process may also provide a versatile and quick method to prepare biomass-based biosorbents with high adsorption capabilities. 

## Figures and Tables

**Table 1:** BET surface areas and total pore volumes of the biosorbents

Biosorbents	BET Surface Area (m^2^/g)	Total Pore Volume (cm^3^/g)
HGP	4.12	0.0335
MGP	3.01	0.0193

**Table 2:** Constants of Langmuir and Freundlich simulations of MB adsorption isotherms.

Biosorbent	Langmuir Isotherm Constants			Freundlich Isotherm Constants		
	*k*_3_ (L/mg)	*q_m_* (mg/g)	*R*^2^	1/*n* (g/L)	*k*_4_ (mg/g)	*R*^2^
MGP	0.33	215.7	0.994	0.45	6.06	0.189
HGP	0.16	192.7	0.991	0.33	5.32	0.454

**Table 3:** Pseudo-first and pseudo-second order kinetic parameters for MB adsorption on the MGP and HGP biosorbents.

Sample	*q_e,exp._* (mg/g)	Pseudo-First Order			Pseudo-Second Order		
		*k*_5_ (1/min)	*q_e_cal._* (mg/g)	*R*^2^	*k*_6_ (g/mg/min)	*q_e_cal._* (mg/g)	*R*^2^
MGP	208.1	0.027	203.3	0.931	4.2 × 10^−4^	217.9	0.997
HGP	183.9	0.024	182.6	0.990	7.9 × 10^−5^	240.4	0.994

**Table 4:** Comparison of the maximum monolayer adsorption of MB onto various biosorbents.

Biosorbent	*q_m_* (mg/g)	Ref.
Grape-peel	215.7	This work
Platanus orientalis leaf	114.9	[25]
Tea waste	85.2	[28]
Peanut hull	68.0	[2]
Garlic peel	82.6	[16]
Banana peel	20.8	[29]
Orange peel	18.6	[29]
Spent coffee grounds	18.7	[30]
Rice husk	40.6	[3]
Grape pulp	118.3	[23]
